# Supplementary material for: Effective Connectivity Evaluation of Resting-State Brain Networks in Alzheimer’s Disease, Amnestic Mild Cognitive Impairment, and Normal Aging: An Exploratory Study
Source: Brain Sci. 2023 Feb 4;13(2):265. doi: 10.3390/brainsci13020265 (PMC9954618; doi:10.3390/brainsci13020265)
Supplement: Supplementary file 1 [file brainsci-13-00265-s001.zip › brainsci-2150243-supplementary.pdf]

Table S1. The MNI coordinates associated with the 32 regions of interest from 8 resting-state network

Resting-state network	Region	Region (abbreviation)	Coordination
Default mode network	Medial prefrontal cortex	MPFC	1 55 -3
Default mode network	Left lateral parietal	LP(L)	-39 -77 33
Default mode network	Right lateral parietal	LP(R)	47 -67 29
Default mode network	Posterior cingulate cortex	PCC	1 -61 38
Sensorimotor network	Left lateral sensorimotor	Sensorimotor lateral(L)	-55 -12 29
Sensorimotor network	Right lateral sensorimotor	Sensorimotor lateral(R)	56 -10 29
Sensorimotor network	Superior sensorimotor	Sensorimotor superior	0 -31 67
Visual network	Medial visual	Visual medial	2 -79 12
Visual network	Occipital visual	Visual occipital	0 -93 -4
Visual network	Left lateral visual	Visual lateral(L)	-37 -79 10
Visual network	Right lateral visual	Visual lateral(R)	38 -72 13
Salience network	Anterior cingulate cortex	ACC	0 22 35
Salience network	Left anterior insula	Alnsula(L)	-44 13 1
Salience network	Right anterior insula	Alnsula(R)	47 14 0
Salience network	Left rostral prefrontal cortex	RPFC(L)	-32 45 27
Salience network	Right rostral prefrontal cortex	RPFC(R)	32 46 27
Salience network	Left supramarginal gyrus	SMG(L)	-60 -39 31
Salience network	Right supramarginal gyrus	SMG(R)	62 -35 32
Dorsal attention network	Left frontal eye field	FEF(L)	-27 -9 64
Dorsal attention network	Right frontal eye field	FEF(R)	30 -6 64
Dorsal attention network	Left intraparietal sulcus	IPS(L)	-39 43 52
Dorsal attention network	Right intraparietal sulcus	IPS(R)	39 -42 54
Frontopariatal network	Left lateral prefrontal cortex	LPFC(L)	-43 33 28
Frontopariatal network	Left posterior parietal cortex	PPC(L)	-46 -58 49
Frontopariatal network	Right lateral prefrontal cortex	LPFC(R)	41 38 30
Frontopariatal network	Right posterior parietal cortex	PPC(R)	52 -52 45
Language network	Left inferior frontal gyrus	IFG(L)	-51 26 2
Language network	Right inferior frontal gyrus	IFG(R)	54 28 1
Language network	Left posterior superior temporal gyrus	pSTG(L)	-57 -47 15

Language network	Right posterior superior temporal gyrus	pSTG(R)	59 -42 13
Cerebellar network	Anterior cerebellar	CA	0 -63 -30
Cerebellar network	Posterior cerebellar	CP	0 -79 -32

Table S2. Mean/ standard deviation (STD) of connection strengths in the default mode network. The rows of the table show source regions, and columns represent target regions. Connections with power > 0.05 Hertz (Hz) are shown in bold. The Mean/STD of connection strengths in the resting-state network are presented in Hz.

**Default mode network Late-onset AD**				
**Mean/STD**	**MPFC**	**LP(L)**	**LP(R)**	**PCC**
**MPFC**	-0.1948±0.5031	-0.049±0.4421	**0.0597±0.3906**	**0.0702±0.3805**
**LP(L)**	-0.1917±0.4564	0.241±0.3139	0.0376±0.2672	0.0479±0.3619
**LP(R)**	-0.2838±0.5346	0.0096±0.3236	0.1762±0.2936	**0.1237±0.327**
**PCC**	-0.1744±0.4374	-0.1018±0.408	**0.1993±0.4116**	0.152±0.3759
**Default mode network aMCI**				
**Mean/STD**	**MPFC**	**LP(L)**	**LP(R)**	**PCC**
**MPFC**	-0.0939±0.2138	-0.0971±0.5526	**0.0744±0.5253**	-0.2802±0.5441
**LP(L)**	-0.1103±0.2917	0.1456±0.5393	**0.0714±0.4277**	**0.0933±0.43**
**LP(R)**	-0.1244±0.4472	-0.2282±0.5024	0.1601±0.4274	**0.1493±0.3947**
**PCC**	**0.1298±0.288**	-0.2154±0.4811	-0.0417±0.5369	-0.2016±0.5045
**Default mode network normal**				
**Mean/STD**	**MPFC**	**LP(L)**	**LP(R)**	**PCC**
**MPFC**	-0.1123±0.3571	-0.3602±0.6139	-0.2347±0.5749	0.0303±0.3984
**LP(L)**	**0.173±0.3913**	-0.0803±0.4216	**0.1519±0.3891**	-0.0314±0.3136
**LP(R)**	0.0289±0.4336	**0.1357±0.5376**	0.1074±0.422	**0.1114±0.3123**
**PCC**	0.018±0.3286	-0.1799±0.6781	**0.278±0.2993**	0.0687±0.2975

The medial prefrontal cortex (MPFC), left lateral parietal (LP(L)), right lateral parietal (LP(R)), and posterior cingulate cortex (PCC).

Table S3. Mean/ standard deviation (STD) of connection strengths in the cerebellar network. The rows of the table show source regions, and columns represent target regions. Connections with power > 0.05 Hertz (Hz) are shown in bold. The Mean/STD of connection strengths in the resting-state network are presented in Hz.

**Cerebellar network Late-onset AD**		
**Mean/STD**	**CA**	**CP**
**CA**	0.5268±0.3538	-0.1225±0.3036
**CP**	**0.1576±0.3913**	0.2551±0.2948
**Cerebellar network aMCI**		
**Mean/STD**	**CA**	**CP**
**CA**	0.1126±0.6022	-0.1354±0.3889
**CP**	-0.0354±0.8016	0.0028±0.4616
**Cerebellar network normal**		
**Mean/STD**	**CA**	**CP**

**CA**	0.1448±0.6776	0.0308±0.4729
**CP**	-0.2734±0.7969	0.058±0.4355

Cerebellar Anterior (CA), Cerebellar Posterior (CP).

Table S4. Mean/ standard deviation (STD) of connection strengths in the dorsal attention network. The rows of the table show source regions, and columns represent target regions. Connections with power > 0.05 Hertz (Hz) are shown in bold. The Mean/STD of connection strengths in the resting-state network are presented in Hz.

**Dorsal attention network Late-onset AD**				
**Mean/STD**	**FEF(L)**	**FEF(R)**	**IPS(L)**	**IPS(R)**
**FEF(L)**	-0.0519±0.5304	**0.2034±0.5523**	**0.287±0.411**	-0.1844±0.5695
**FEF(R)**	**0.1537±0.2694**	0.0327±0.3593	0.0364±0.6034	-0.0936±0.4363
**IPS(L)**	-0.1418±0.5812	-0.2926±0.6407	-0.0756±0.554	-0.2425±0.7862
**IPS(R)**	-0.1984±0.6116	0.0457±0.5509	0.0264±0.2732	-0.0185±0.4051
**Dorsal attention network aMCI**				
**Mean/STD**	**FEF(L)**	**FEF(R)**	**IPS(L)**	**IPS(R)**
**FEF(L)**	0.0979±0.4236	**0.0864±0.4087**	-0.2168±0.5523	-0.0235±0.4393
**FEF(R)**	0.0182±0.5701	-0.1093±0.4494	-0.372±0.5519	-0.1774±0.5737
**IPS(L)**	-0.0171±0.3375	**0.138±0.5039**	-0.0658±0.5152	**0.0961±0.2959**
**IPS(R)**	-0.0324±0.4299	**0.1551±0.4253**	**0.0762±0.5769**	0.0954±0.4449
**Dorsal attention network normal**				
**Mean/STD**	**FEF(L)**	**FEF(R)**	**IPS(L)**	**IPS(R)**
**FEF(L)**	0.0238±0.4555	**0.1554±0.4249**	-0.0836±0.4543	0.0371±0.5227
**FEF(R)**	**0.1223±0.5017**	0.034±0.3776	-0.1564±0.5864	**0.1095±0.4451**
**IPS(L)**	0.0048±0.36	-0.0178±0.3541	0.0609±0.3527	**0.1062±0.4176**
**IPS(R)**	-0.3116±0.3849	0.0003±0.2841	-0.0355±0.5638	0.1137±0.4163

Left frontal eye field (FEF(L)), right frontal eye field (FEF(R)), left intraparietal sulcus (IPS(L)), right intraparietal sulcus (IPS(R)).

Table S5. Mean/ standard deviation (STD) of connection strengths in the sensorimotor network. The rows of the table show source regions, and columns represent target regions. Connections with power > 0.05 Hertz (Hz) are shown in bold. The Mean/STD of connection strengths in the resting-state network is presented in Hz.

**Sensorimotor network Late-onset AD**			
**Mean/STD**	**SML(L)**	**SML(R)**	**SMS**
**SML(L)**	0.0062±0.6041	-0.2204±0.7098	-0.1655±0.4555
**SML(R)**	**0.1037±0.3392**	0.2294±0.4347	-0.2055±0.4804
**SMS**	-0.2257±0.494	-0.0743±0.5211	-0.0737±0.4367
**Sensorimotor network aMCI**			
**Mean/STD**	**SML(L)**	**SML(R)**	**SMS**
**SML(L)**	0.0432±0.4886	**0.0994±0.4121**	-0.1453±0.3633
**SML(R)**	0.0078±0.621	0.1664±0.3402	-0.4553±0.5763
**SMS**	-0.0601±0.5495	**0.0602±0.4092**	-0.1174±0.3768
**Sensorimotor network normal**			

Mean/STD	SML(L)	SML(R)	SMS
**SML(L)**	0.1798±0.3148	**0.083±0.3874**	-0.2487±0.5767
**SML(R)**	**0.2023±0.5704**	0.1763±0.4755	-0.1815±0.4629
**SMS**	-0.0538±0.5564	-0.1753±0.7828	-0.1277±0.3987

Right sensorimotor lateral (SML(R)), left sensorimotor lateral (SML(L)), sensorimotor superior (SMS).

Table S6. Mean/ standard deviation (STD) of connection strengths in the visual network. The rows of the table show source regions, and columns represent target regions. Connections with power > 0.05 Hertz (Hz) are shown in bold. The Mean/STD of connection strengths in the resting-state network is presented in Hz.

**Visual network Late-onset AD**				
**Mean/STD**	**VM**	**VO**	**VL(L)**	**VL(R)**
**VM**	-0.0424±0.3695	-0.0446±0.3921	-0.3499±0.5886	-0.0059±0.6237
**VO**	**0.2141±0.4857**	-0.0694±0.38	-0.0142±0.524	-0.0347±0.5984
**VL(L)**	**0.1123±0.189**	-0.048±0.3396	0.2159±0.3503	**0.1616±0.3396**
**VL(R)**	**0.1078±0.2355**	0.0385±0.3839	-0.0735±0.5119	0.1482±0.4724
**Visual network aMCI**				
**Mean/STD**	**VM**	**VO**	**VL(L)**	**VL(R)**
**VM**	-0.0316±0.3711	-0.1772±0.3558	-0.1024±0.6743	-0.1621±0.4861
**VO**	**0.4176±0.386**	-0.2015±0.4258	-0.2809±0.5072	0.0259±0.4511
**VL(L)**	0.0186±0.3126	0.0323±0.4403	0.0516±0.3958	**0.1064±0.3256**
**VL(R)**	**0.1545±0.204**	-0.1764±0.347	**0.1657±0.4102**	0.3012±0.3156
**Visual network normal**				
**Mean/STD**	**VM**	**VO**	**VL(L)**	**VL(R)**
**VM**	-0.1311±0.3386	-0.0486±0.4331	0.0279±0.4028	-0.3062±0.6314
**VO**	**0.4184±0.404**	-0.1143±0.3851	**0.091±0.6097**	-0.3243±0.5777
**VL(L)**	**0.1847±0.3356**	-0.1349±0.3413	0.3786±0.3576	-0.0093±0.3689
**VL(R)**	**0.0966±0.3162**	-0.0522±0.4088	**0.1251±0.2682**	0.2283±0.4745

Right visual lateral (VL(R)), left visual lateral (VL(L)), visual medial (VM), visual occipital (VO).

Table S7. Mean/ standard deviation (STD) of connection strengths in the salience network. The rows of the table show source regions, and columns represent target regions. Connections with power > 0.05 Hertz (Hz) are shown in bold. The Mean/STD of connection strengths in the resting-state network is presented in Hz.

**Salience network Late-onset AD**							
**Mean/STD**	**ACC**	**AInsula(L)**	**AInsula(R)**	**RPFC(L)**	**RPFC(R)**	**SMG(L)**	**SMG(R)**
ACC	-0.0685±0.3314	**0.0734±0.3332**	-0.1126±0.4785	-0.053±0.2434	**0.1563±0.3788**	0.0338±0.3909	-0.191±0.3693
AInsula(L)	-0.2551±0.3911	-0.1003±0.2954	**0.1431±0.4105**	0.0025±0.3514	-0.1262±0.3621	-0.2758±0.2536	-0.178±0.3188

AInsula(R)	-0.2048±0.3178	0.0493±0.4832	-0.2169±0.3835	-0.2599±0.4567	-0.0179±0.4487	-0.2567±0.3925	-0.1413±0.3711
RPFC(L)	**0.0604±0.2809**	0.0036±0.3916	-0.0552±0.2359	0.1224±0.2828	**0.254±0.3625**	-0.0201±0.271	-0.0471±0.2209
RPFC(R)	**0.1266±0.4422**	-0.1048±0.3229	-0.0006±0.3904	-0.0056±0.342	-0.0021±0.3345	-0.1394±0.3698	-0.0685±0.3065
SMG(L)	-0.0832±0.5319	**0.1334±0.2185**	-0.2121±0.2874	-0.1108±0.1582	-0.1079±0.3271	-0.0477±0.2394	**0.1198±0.3465**
SMG(R)	**0.1019±0.3924**	-0.0541±0.3032	0.049±0.2141	-0.0629±0.237	-0.1004±0.3067	**0.1471±0.3906**	0.0765±0.3364
**Salience network aMCI**							
**Mean/STD**	**ACC**	**AInsula(L)**	**AInsula(R)**	**RPFC(L)**	**RPFC(R)**	**SMG(L)**	**SMG(R)**
ACC	0.0756±0.2328	0.0402±0.3674	-0.0424±0.3365	0.0079±0.3286	0.0104±0.4143	-0.0459±0.2523	**0.0737±0.2865**
AInsula(L)	-0.1768±0.2821	-0.1455±0.4154	0.0322±0.5298	-0.0726±0.3978	-0.0487±0.4519	-0.3316±0.6011	-0.1083±0.4297
AInsula(R)	-0.1238±0.4495	0.0804±0.41	-0.0279±0.3851	-0.2344±0.5008	**0.065±0.2994**	-0.0894±0.6053	-0.0963±0.4094
RPFC(L)	**0.0967±0.3008**	**0.1018±0.3191**	-0.156±0.2631	-0.0502±0.3451	**0.3015±0.3969**	-0.0837±0.3707	0.0188±0.2798
RPFC(R)	0.0473±0.3658	0.0158±0.313	-0.0073±0.3187	-0.1013±0.3716	-0.1358±0.3231	-0.024±0.5366	0.0156±0.3421
SMG(L)	-0.0228±0.1864	-0.0102±0.2231	0.0315±0.3879	-0.0972±0.2842	0.0063±0.3246	-0.1159±0.488	0.0278±0.4431
SMG(R)	0.0019±0.2925	**0.0934±0.2666**	-0.044±0.2739	-0.0789±0.3724	-0.0823±0.2394	0.032±0.2958	0.0808±0.3194
**Salience network normal**							
**Mean/STD**	**ACC**	**AInsula(L)**	**AInsula(R)**	**RPFC(L)**	**RPFC(R)**	**SMG(L)**	**SMG(R)**
ACC	0.0898±0.3346	-0.0789±0.264	-0.0558±0.2466	-0.0015±0.4281	-0.0043±0.2929	-0.0894±0.3328	0.0299±0.3538

AInsula(L)	-0.0742±0.5928	-0.1069±0.3336	**0.1857±0.4851**	-0.2104±0.358	0.0026±0.3867	-0.11±0.3981	**0.1172±0.6132**
AInsula(R)	-0.1137±0.4138	**0.2811±0.3675**	-0.1316±0.3279	-0.0593±0.3459	0.0003±0.2478	-0.0754±0.3636	-0.0004±0.4806
RPFC(L)	0.0093±0.4258	-0.0049±0.359	-0.1526±0.3922	0.0711±0.2513	0.0379±0.4475	-0.1467±0.4117	-0.166±0.3441
RPFC(R)	-0.043±0.3404	-0.0778±0.2644	-0.0394±0.4763	**0.0517±0.4031**	0.0326±0.4074	-0.304±0.5116	-0.0432±0.3452
SMG(L)	0.0475±0.2129	0.025±0.2584	-0.0115±0.1812	0.0306±0.2879	-0.0272±0.2153	0.1568±0.3673	**0.237±0.2056**
SMG(R)	0.033±0.2942	-0.0927±0.2455	-0.0227±0.3366	0.0381±0.4073	0.02±0.3302	0.0138±0.3506	0.0764±0.3547

Anterior cingulate cortex (ACC), left anterior insula (AInsula(L)), right anterior insula (AInsula(R)), left rostral prefrontal cortex (RPFC(L)), right rostral prefrontal cortex (RPFC(R)), left supramarginal gyrus (SMG(L)), right supramarginal gyrus (SMG(R)).

Table S8. Mean/ standard deviation (STD) of connection strengths in the language network. The rows of the table show source regions, and columns represent target regions. Connections with power > 0.05 Hertz (Hz) are shown in bold. The Mean/STD of connection strengths in the resting-state network is presented in Hz.

**Language network Late-onset AD**				
**Mean/STD**	**IFG(L)**	**IFG(R)**	**pSTG(L)**	**pSTG(R)**
IFG(L)	0.2277±0.2982	**0.2153±0.4016**	-0.4911±0.3282	-0.3382±0.3775
IFG(R)	-0.0686±0.3032	0.1019±0.4515	-0.6058±0.671	-0.4233±0.3589
pSTG(L)	**0.269±0.1682**	**0.1327±0.3245**	-0.0395±0.4104	**0.2552±0.3479**
pSTG(R)	**0.1718±0.3441**	**0.1447±0.2669**	**0.3382±0.4182**	-0.0672±0.4418
**Language network aMCI**				
**Mean/STD**	**IFG(L)**	**IFG(R)**	**pSTG(L)**	**pSTG(R)**
IFG(L)	-0.076±0.2187	0.0397±0.6772	-0.1321±0.4353	-0.1396±0.5338
IFG(R)	**0.0556±0.3933**	-0.2367±0.3432	-0.2553±0.5652	-0.1733±0.3716
pSTG(L)	**0.1992±0.2714**	-0.1669±0.3677	0.0728±0.4527	**0.0688±0.3189**
pSTG(R)	0.025±0.2168	-0.0056±0.48	**0.093±0.4245**	0.0532±0.3556
**Language network normal**				
**Mean/STD**	**IFG(L)**	**IFG(R)**	**pSTG(L)**	**pSTG(R)**
IFG(L)	-0.1363±0.3306	**0.1102±0.5921**	-0.314±0.3783	-0.159±0.6939
IFG(R)	-0.064±0.4753	0.0773±0.3986	-0.2375±0.3974	-0.1185±0.3843
pSTG(L)	**0.0689±0.2677**	0.0296±0.3855	0.1153±0.3405	**0.2704±0.4178**
pSTG(R)	**0.0895±0.3506**	-0.0159±0.4873	**0.1547±0.4383**	-0.1758±0.417

Left inferior frontal gyrus (IFG(L)), right inferior frontal gyrus (IFG(R)), left posterior superior temporal gyrus (pSTG(L)), right posterior superior temporal gyrus (pSTG(R)).

Table S9. Mean/ standard deviation (STD) of connection strengths in the frontoparietal network. The rows of the table show source regions, and columns represent target regions. Connections with power > 0.05 Hertz (Hz) are shown in bold. The Mean/STD of connection strengths in the resting-state network is presented in Hz.

**Frontoparietal network Late-onset AD**				
**Mean/STD**	**LPFC(L)**	**PPC(L)**	**LPFC(R)**	**PPC(R)**
LPFC(L)	0.1158±0.3742	-0.1073±0.3525	**0.1378±0.2791**	-0.0848±0.912
PPC(L)	0.0014±0.4017	0.1144±0.4278	-0.0895±0.4719	**0.3607±0.377**
LPFC(R)	**0.1584±0.2611**	-0.0867±0.3348	0.1609±0.3069	-0.2101±0.527
PPC(R)	-0.1621±0.5642	0.0068±0.4752	-0.135±0.4989	-0.333±0.3608
**Frontoparietal network aMCI**				
**Mean/STD**	**LPFC(L)**	**PPC(L)**	**LPFC(R)**	**PPC(R)**
LPFC(L)	-0.1075±0.3744	0.0424±0.6427	**0.1861±0.562**	-0.2704±0.4646
PPC(L)	0.0443±0.3177	0.0733±0.3901	-0.123±0.3868	**0.2857±0.2411**
LPFC(R)	0.0132±0.4775	-0.0519±0.5009	-0.0091±0.3679	-0.1926±0.5945
PPC(R)	-0.0483±0.4472	0.0125±0.3401	-0.0725±0.4186	0.0929±0.3935
**Frontoparietal network normal**				
**Mean/STD**	**LPFC(L)**	**PPC(L)**	**LPFC(R)**	**PPC(R)**
LPFC(L)	0.0965±0.3387	-0.2764±0.4156	**0.3774±0.2993**	-0.3053±0.6246
PPC(L)	**0.1876±0.2355**	-0.1511±0.3726	-0.0965±0.4852	**0.3863±0.5358**
LPFC(R)	-0.0924±0.4521	-0.323±0.5528	-0.1087±0.5188	-0.2327±0.3933
PPC(R)	-0.07±0.4392	**0.1658±0.2866**	-0.0065±0.7043	-0.095±0.3862

Left lateral prefrontal cortex (LPFC(L)), left posterior parietal cortex (PPC(L)), right lateral prefrontal cortex (LPFC(R)), right posterior parietal cortex (PPC(R)).
